# Assessing Versatility of a Generic End-to-End Platform for IoT Ecosystem Applications

**DOI:** 10.3390/s22030713

**Published:** 2022-01-18

**Authors:** Riccardo Berta, Francesco Bellotti, Alessandro De Gloria, Luca Lazzaroni

**Affiliations:** Department of Electrical, Electronic and Telecommunication Engineering (DITEN), University of Genoa, Via Opera Pia 11A, 16145 Genoa, Italy; berta@elios.unige.it (R.B.); franz@elios.unige.it (F.B.); adg@elios.unige.it (A.D.G.)

**Keywords:** IoT, edge computing, end-to-end systems, development tools, embedded systems and devices

## Abstract

Availability of efficient development tools for data-rich IoT applications is becoming ever more important. Such tools should support cross-platform deployment and seamless and effective applicability in a variety of domains. In this view, we assessed the versatility of an edge-to-cloud system featuring Measurify, a framework for managing smart things. The framework exposes to developers a set of measurement-oriented resources that can be used in different contexts. The tool has been assessed in the development of end-to-end IoT applications in six Electronic and Information Technologies Engineering BSc theses that have highlighted the potential of such a system, both from a didactic and a professional point of view. The main design abstractions of the system (i.e., generic sensor configuration, simple language with chainable operations for processing data on the edge, seamless WiFi/GSM communication) allowed developers to be productive and focus on the application requirements and the high-level design choices needed to define the edge system (microcontroller and its sensors), avoiding the large set-up times necessary to start a solution from scratch. The experience also highlighted some usability issues that will be addressed in an upcoming release of the system.

## 1. Introduction

In the emerging Internet of Things (IoT) paradigm, data collected from the field fuel a variety of applications (e.g., monitoring, prediction, maintenance, etc.) in multiple domains [1]. An IoT ecosystem involves two major sides: the edge and the cloud. Cloud services are needed to support data access and management. These services are typically developed and maintained through platform-as-a-service frameworks. Edge devices, on the other hand, are getting ever more relevant as fully integrated tools in a seamless computation continuum from the field to the cloud. Executing code in proximity to data sources aims at reducing latency, energy consumption, and bandwidth occupation [2]. The generic edge device term involves a variety of devices, ranging from FPGAs and devices with few KBs of memory to families of microcontrollers (e.g., [3]), smartphones, and high-performance Machine Learning (ML)-enabled microcontrollers (e.g., the Coral Dev Board [4]). In order to increase scalability, the IoT paradigm promotes a hierarchical structure of connected devices, dynamically organizing the data processing to optimize overall resource consumption. 

In this context, there is a growing need to provide developers with tools able to support efficient design and implementation of applications, allowing them to focus on the application logic rather than on the implementation details (e.g., about the management of connection and delivery of information packets, organization of the data processing across various stages from the field to the cloud). Despite the use of common components (e.g., databases, application programming interfaces, protocols), the development and deployment process is challenging and time-consuming. 

The availability of efficient application development tools is key to the success of any digital ecosystem. Commercial companies (e.g., Amazon, Microsoft, Google) have established efficient IoT ecosystems based on powerful cloud services, but they rely on proprietary technologies, with very limited interoperability and development opportunities for third parties.

In this context, we are interested in analyzing the research question about the ability of a non-vendor-locked, interoperable framework to support effective and efficient development for a variety of IoT applications in different application domains. Versatility is very important both from a didactic (students can be exposed to and practice with very different use cases) and business (a company, especially small/medium, could rapidly prototype/develop a wide application portfolio) point of view. Thus, our analysis also investigates what the main design abstractions are in order to support cross-application-domain versatility. This also involves understanding the knowledge level needed from developers using such a tool.

In this paper, we address this question exploiting Measurify, a cloud-based (but not vendor-locked), open-source, measurement-oriented framework, for managing smart things in IoT ecosystems. Measurify includes Edgine (Edge engine), a cross-platform edge computing system designed to support developers building the edge side of IoT applications. Our investigation consisted of assessing the versatility of Measurify in the development of a set of apps in a variety of domains (business, environment, and sport). As developer users for our analysis, we chose Electronic and Information Technology Engineering BSc students doing their final thesis. This target on one hand intends to assess the simplicity of development (given the elementary professional level of the subjects), on the other hand it stresses the importance of didactics (and didactic tools) to allow growing a new generation of electronic system designers with hands-on experience in end-to-end IoT applications (and not “only” on specific aspects).

The remainder of the manuscript is organized as follows. Section 2 gives an outlook of the state of the art. Section 3 presents the Measurify and Edgine platforms that manage the cloud and edge side, respectively. Section 4 presents a set of IoT applications developed with the proposed system in three main areas: business, environment, and sports. In Section 5, results are analyzed and discussed. Section 6 draws the conclusions and proposes possible directions for future works.

## 2. Related Work

Edge devices have become increasingly important in the Internet of Things (IoT) scenario [1] as fully integrated instruments in a continuously operating computing flow from the field to the cloud. Such an edge computing paradigm [5], which moves the computation (including sophisticated artificial intelligence (AI)) as close as possible to the data sources [2], aims at reducing latency, energy consumption, and bandwidth usage. 

Given the vast application potential, industry giants are deeply engaged in developing hardware and software solutions in the field. Google released the Edge Tensor Processing Unit (TPU) [6] and Cloud IoT Edge [7]. The former is an application-specific integrated circuit (ASIC) created specifically to run AI at a peripheral level, while the latter is an edge computing platform that extends the capabilities of Google Cloud data processing and Machine Learning (ML) to edge devices. The idea is to build AI models on the cloud, then use them on IoT edge cloud devices by exploiting the potential offered by the Edge TPU hardware accelerator. This circuit is also able to run TensorFlow Lite [8], a platform that provides a set of tools allowing the user to convert TensorFlow [9] neural network (NN) models into simplified and reduced versions, suitable for edge devices. Recently, a further reduced version of TensorFlow Lite has been released, namely, TensorFlow Lite Micro, which is specifically designed to run ML models on digital signal processors (DSPs), microcontrollers, and other devices with limited memory [10].

Within its offer of cloud services (AWS), Amazon provides the IoT solution Greengrass [11], which simplifies the inference of ML locally on devices through archetypes created, trained, and optimized in the cloud. The AWS IoT Greengrass has the Lambda runtime [12], which is a serverless computation service that allows running code without provisioning or managing any infrastructure, automatically managing the underlying compute resources. The minimum hardware requirements are 1 GHz processor frequency and 128 MB of RAM.

Microsoft provides Azure IoT Edge [13], a service that allows distributing cloud workloads and running them on IoT peripheral devices. Latency is reduced as the data is processed locally, with the possibility of using Microsoft’s Project Brainwave [14], a deep learning platform for real-time AI inference in the cloud and on the edge. Peripheral devices can also work in conditions of poor Internet connection, thanks to Azure device management that automatically synchronizes the most recent status of the devices after reconnecting to the network. IoT Edge supports numerous languages, including C, C#, Java, Node.js, and Python. Microsoft also released EdgeML [15], a suite of ML algorithms designed for deployment in low-resource contexts. The published results on the use of EdgeML for training in the cloud in conditions of limited computing power indicate the quality of the project [16,17,18,19,20,21].

IBM developed Edge Application Manager [22], an intelligent, secure, and flexible platform that provides a management tool for edge processing. The proposed solution is autonomous, i.e., it allows a single administrator to manage scale, variability, and frequency of change of application environments across endpoints simultaneously. Edge endpoints run on Red Hat Open-Shift [23] containers. IBM Edge Application Manager also supports AI tools for deep learning and voice and image recognition, as well as video and acoustics analysis.

Considering cross-platform support, computational resource allocation algorithms have been developed in [24] in order to improve the performance of vehicular networks, which is a key IoT application. The system uses the k-nearest neighbor (kNN) algorithm for selecting the execution platform (e.g., cloud computing, mobile edge computing, or local computing), and reinforcement learning (RL) for the resources allocation task. The simulation results show that, compared to the basic algorithm in which all activities are performed on the local or mobile edge computing server, the resource allocation scheme allows a significant latency reduction by around 80%.

To deal with the problem of energy consumption of IoT devices, [25] suggests virtualization, particularly container-based virtualization, which also enables handling the multi-platform and multi-OS challenge. In this context, [26] presents a performance evaluation study that shows the strengths and weaknesses of several low-power devices when handling container-virtualized instances opposite to native executions.

Due to its flexibility and the tight connection to IoT devices, edge computing embodies several very different use-cases. Debauche et al. [27] propose this approach to exploit a modular climatic enclosure through IoT devices virtualization. This facilitates the exploitation of common semantic rules for each user about a common use-case. The Docker container technology is used to deploy the needed software on the local device [28]. A cloud interface is also used to collect data coming from local installations and visualize them. A similar approach is employed in [29], which investigates a smart IoT-based method for firefighting, collecting data coming from heterogeneous sources (i.e., drones, wearable technologies, etc.). As the system’s reactive speed is crucial for this kind of task, a simulation is performed in this perspective, which results in a 50% improvement in system latency. On the other hand, [30] focuses on edge video surveillance. In detail, it is shown how, when the system has a failure, maintenance takes a long time to locate, while faulty data waste storage space on the cloud. The proposed model makes a real-time assessment of the usefulness of the video data and warns the end-users of possible failures. In this way, faulty video data are directly handled on the edge rather than being uselessly uploaded to the cloud. Another edge computing-based architecture was developed in the air pollution monitoring system presented in [31]. The system’s sensors gather the air quality data in real-time and transmit it to an edge device that processes and analyzes them. The prototype works on Arduino boards and relies on the IBM Watson IoT platform [32]. The final model reduces the computational burden over sensing nodes up to 70%. In [33], an IoT-based manufacturing context following the edge computing approach is analyzed. Results show that this paradigm, compared to traditional approaches (i.e., cloud-based), provides bandwidth optimization, real-time operation, and increased agility and security levels. The authors of [34] propose an edge computing simulation framework for IoT heterogeneous devices, which allows users to test their application in an easy and configurable manner. The simulator is able to model various features, including device heterogeneity, application composition, a variety of IoT communication protocols, device movement and mobility, and battery. This allows developers to test their prototypes and identify criticalities in an end-to-end approach.

Table 1 summarizes and compares the above presented edge-computing systems. We do not know whether these systems exploit specific features of the cloud service on which they are hosted, as this is not specified in the corresponding papers.

In this wide landscape, the proposed Edgine framework aims at offering a solution for efficient development, with two main perspectives. On the one hand, it intends to abstract as much as possible from the application domain (e.g., [35]) to foster the re-use of code and knowledge. On the other hand, it is built exclusively on open-source and cloud/edge-provider independent technologies. This aims at quickly developing fully portable applications, which is a key advantage in the rapidly evolving IoT world. To summarize, cross-domain versatility is a major goal of our research, which does not look to be a major concern of other studies.

## 3. Materials and Methods

An IoT system relying on the edge computing paradigm typically involves three main components:

- a field device that collects data from the surrounding environment via sensors;
- an execution engine, operating on the field, capable of interpreting and processing the field data in order to send to the cloud only higher-level, relevant information;
- a cloud server that collects data from the periphery. Third-party applications interact with the server in order to manage the available field execution engines and to support data queries by the analysts.

This article presents a generic, flexible architecture that we designed to implement an end-to-end system that could be deployed to enable the IoT paradigm in a variety of application domains. This section focuses on the architecture of the cloud server (namely, Measurify) and the field execution engine (namely, Edgine). In the next section, while describing our operational experience with a set of real-world applications, we will indicate some field devices that have been used and configured to collect data from the field.

### 3.1. Measurify Cloud API

Measurify is a cloud-based, abstract, and measurement-oriented platform we developed to manage intelligent objects in IoT ecosystems. Measurify models these objects as web resources by exposing them through APIs that respect a Representational State Transfer (REST) architecture. In this way, remote access to data and resources occurs through a platform-independent HTTPS interface, which supports the development of applications that make use of these objects. Table 2 provides an outlook of the main Measurify’s resources. More details can be found in [36].

**Figure 1:**
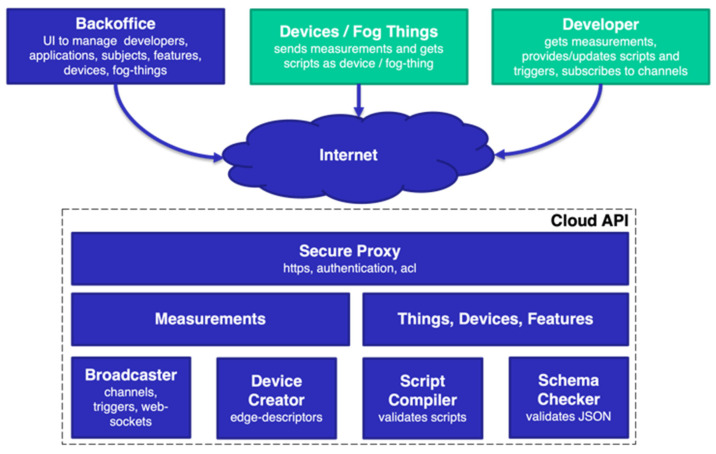
Measurify cloud API architecture.

Not only does Measurify collect all the information sent from the field by an Edgine instance, but it also provides the interface to the developer for remotely programming a deployed field execution engine (Edgine). Each Edgine instance can access a tenant space within a Measurify cloud installation by providing its credentials. If authenticated by Measurify, the Edgine will receive a JSON Web Token (JWT), which must be inserted in the header of all subsequent HTTPS requests to guarantee the authorization. The high-level block diagram of the overall edge-cloud system is summarized in Figure 1, which shows both the configuration and the execution phases of a generic IoT eco-system application supported by the Measurify platform. In the configuration phase, the developer specifies things, devices, features, user roles/rights, and scripts pertaining to the new application. In the execution phase, the devices collect, process, and send measurements to the cloud that can be queried by authorized users. The two phases are not mutually exclusive in terms of time, and, for instance, a developer may add new things, features, scripts, etc., also during the execution.

Measurify relies on the MongoDB [37] and Node.js [38] open source technologies. More details on the implementation can be found in [36].

### 3.2. Edgine Runtime System

Edgine is a cross-platform edge system able to parse from the cloud and locally run scripts associated with (some of) its available resources. The idea is to have a set of generic, limited-resource devices that are deployed in the field and can be dynamically programmed by a remote developer/user through a cloud server (namely, Measurify). This allows to seamlessly exploit a set of field-deployed devices for various kinds of tasks, even in different application domains. In an edge-to-cloud continuum computing perspective, the Edgine abstraction has been designed for remote system configuration in terms of settings (e.g., configuration of the available sensors) and executable scripts. 

**Figure 2:**
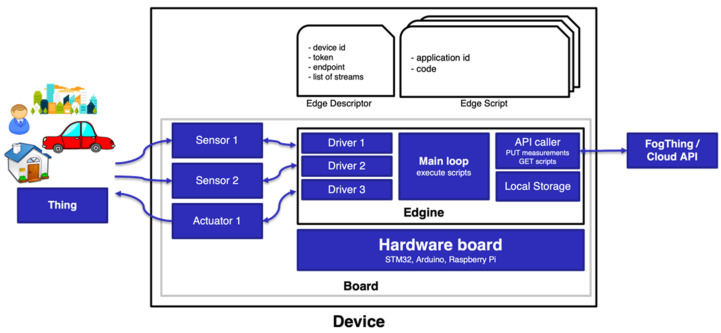
Block diagram of a field device.

**Figure 3:**
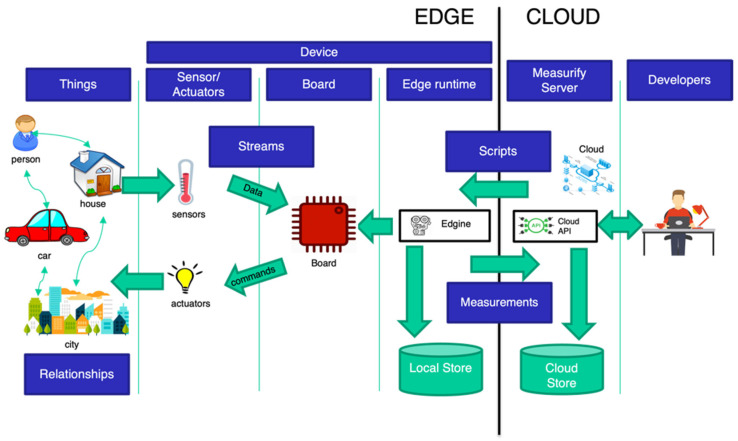
Edgine–Measurify overall end-to-end system architecture.

The Edgine’s runtime lifecycle consists of two parts: an initialization and a continuous loop. In the start-up, Edgine connects to the API to download its description, in particular the list of scripts to be executed and the parameter values for its configuration. During the loop, Edgine executes each assigned script in sequence. Data are then sent through a POST request on the dedicated cloud API route, providing, in the body of the request, also traceability information such as the identification of the device, the script that generated it, and a timestamp. During the execution, malfunctions may occur that can be reported in detail to the cloud. In case of network failures/issues, data are locally stored in a buffer to be sent again when the connection returns stable. Figure 2 shows a block diagram of a field device executing an Edgine module and communicating with a Measurify server. The picture shows that the device performs field measurements in an environment (characterized by one or more things). The device does this by executing a script downloaded from the server. Before the executable instructions, the device had to download a descriptor specifying the usable sensors, the communication parameters, and the executable script(s), as we will see in the next paragraph. Table 3 synthesizes the HTTP requests during the two phases of start-up (authentication and download of the scripts) and infinite loop (upload of the measurements). A schematic structure of the Edgine–Measurify overall end-to-end system architecture is depicted in Figure 3. At a high level, the picture shows a downstream (cloud to edge) of commands and an upstream (edge to cloud) of measurements (i.e., data). 

An Edgine’s life cycle is characterized by the execution of a set of macro-processes: access to the cloud; request from the server its (of the Edgine) virtual description; obtain from the server the executable script(s); consequent local configuration; read data from edge sensors; process such data through the downloaded scripts; store processed data on the cloud. 

**Figure 4:**
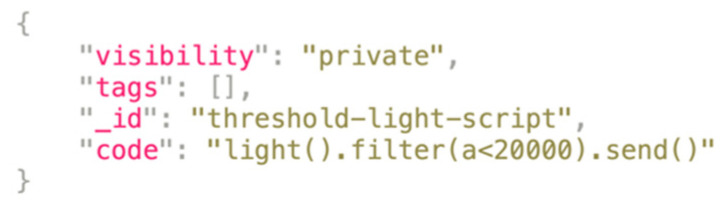
A sample script JSON description.

Thus, after successful authentication, the Edgine requests from the server its own virtual representation. This includes identity, configuration parameters, functionalities, and identifiers of the scripts (script-type resources in Table 2) to be downloaded, parsed, and executed. A script (e.g., Figure 4) is a composition of simple operations whose implementation is preloaded into Edgine. The “code” field of a script document specifies the chain of operations applicable by the Edgine to a given feature’s raw input stream before delivery to the cloud. Each instruction is performed on its input data stream, which is the output of its preceding instruction. The first stage of the chain is applied to raw sensor input data. In the Figure 4 example, the instructions concern the parsing of the available light feature value (expressed in lux), the filtering of all samples whose value is lower than a specific threshold (e.g., 20,000 lux), and the final shipment to Measurify. This example allows the user to monitor lighting in a room. The value on the cloud may then be read, for instance, by a mobile app that notifies the user that the sampled value has fallen below the threshold. At present, the set of supported instructions is reported in Table 4. It appears that the featured set of operations allows basic processing of time-series, avoiding overloading the network and unloading the cloud server as per the edge-computing paradigm [2].

The data upload to the cloud can take place in two possible ways: continuously, i.e., data are sent as soon as they are processed, or in batches, in which case the script specifies the number of measures to be reached so that they are sent in bulk.

Another important aspect that was considered during the development of the Edgine module consists in the communication interface. To this module, we applied the same main criterion as to the overall platform, which is to keep it as abstract as possible from the hardware for the sake of portability. To that end, classes have been created to allow developers to switch from Windows/Linux/macOS PC platforms to Arduino through macros. The main difference between the two platform types concerns the Internet connection. In an Arduino environment, WiFi network connection parameters have to be specified in the code. This is because network connectivity is automatically provided by the system, which is also designed to perform a reconnection in case of signal loss. On the other hand, in PC-type devices, the network connection (and reconnection) is not automated, since a PC user exploits the user interface (UI) of the hosting operating system (OS) to choose, at the system level, among all the available networks. Furthermore, automating the connection would have required different implementations for different OSs, complicating the system. Exploiting such switchable network connection classes, Edgine is now available on the main PC OSs (i.e., Windows, Linux, and macOS) and the following Arduino and Arduino-style boards: Arduino MKR WiFi 1010, Arduino UNO WiFi Rev.2, Arduino NANO 33 IoT, Arduino MKR VIDOR 4000 WiFi, Espressif ESP32-WROVER, and Espressif ESP8266.

## 4. Results

This section intends to assess the ability of the presented toolchain to serve different requirements in quite different application contexts. To this end, we describe six Edgine–Measurify-based real-world applications, covering three major areas: business, environment, and sport. Notably, the applications have been developed by students of the third year of an Electronic and Information Technology Engineering BSc course doing their final thesis, under the supervision of the first author of this paper. This highlights the simplicity of the system, which can also be exploited for real-world designs by technicians with no specific professional experience.

### 4.1. Business Use-Cases

In the following, we describe two use-cases deploying Edgine in the business field. In this sector, real-time performance and the ability to also work off-line (e.g., edge devices installed in remote places in which the connection is unstable) are major advantages with respect to typical cloud computing data flows [39]. 

#### 4.1.1. Shock Monitoring

**Figure 5:**
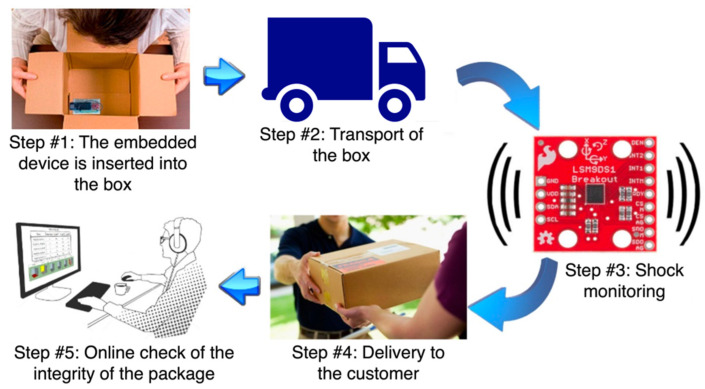
Stages of the shock monitoring system for the transport of goods.

The first application involves the realization of an embedded system for the evaluation of shocks/bumps. The specific use-case considered the logistics and transport sectors, in which it is fundamental to make sure that the shipped goods arrive intact at their destination. The typical steps of the utilization of the proposed system are depicted in Figure 5.

From a developer perspective, the system involves handling two distinct phases:

- the shock monitoring phase, in which data from sensors are retrieved and processed;
- the package integrity check phase, which requires inspecting the history of the detected bumps stored in a database.

The deployed edge system includes a SparkFun 9DoF IMU Breakout-LSM9DS1 sensor, which houses a three-axis accelerometer, a three-axis gyroscope, and a three-axis magnetometer. The sensor is connected to an Arduino MKR WiFi 1010, with a microSD card support, the MKR MEM Shield, to add external memory allowing local storage in case of failures while connecting to the cloud. The LSM9DS1 sensor is connected to the board through the Inter-Integrated Circuit (I2C) serial protocol, whereas the MKR MEM Shield is through a Serial Peripheral Interface (SPI). 

According to the workflow presented in the previous section, the system automatically establishes a connection to a specified WiFi network and logs into Measurify with a username, a password, and a tenant. Then, information regarding the *thing* under measurement is retrieved from the cloud through a GET request, along with scripts to be executed. Measurements are then sent to the server (after local storage, in case of lack of network connectivity). The login and information retrieving phases are performed only once (in the Arduino *setup* function). On the other hand, the sensor data processing and the shipping of the results to the cloud are performed cyclically (in the Arduino *loop* function). Regarding shocks monitoring, the code is inspired by [40], where bidimensional shock detection is used in vehicle collisions to calculate where the car bumped. We adapted it in a three-dimensional way for our use-case. If the magnitude of the shock is higher than the set threshold (named sensitivity), the shock value is sent to Measurify, indicating that a collision occurred. The executable script exploits the *filter* operation (Table 4), so to select over-threshold values only. The point of impact’s location is also computed and sent to Measurify.

**Figure 6:**
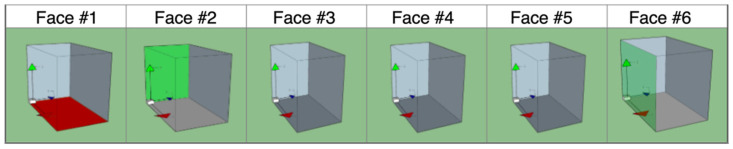
The graphical interface of the shock monitoring web page.

Data visualization for online package integrity check is achieved by designing a web page that features a table reporting the last five impacts and their location (Table 5 shows an example). The location of the impact is inferred from the values of the impact angles in the three dimensions (namely Angle XY, Angle YZ, and Angle XZ). To facilitate understanding, six pictures are provided, each one showing a side of the packaging (Figure 6). The graphical representation shows the intensity of the impact by using a four-key color scale: gray if no impact is detected, green for a slight bump, yellow for a moderate one, and red if an intense shock was detected. The color is set by looking at the impact magnitude value, which is represented in Table 5.

#### 4.1.2. Tank Level Monitoring

**Figure 7:**
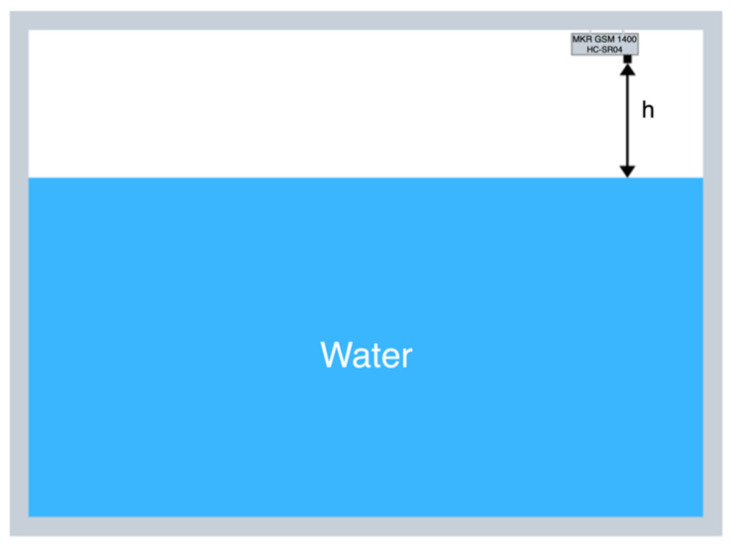
The tank level monitoring system.

The second industrial use-case concerns a system for monitoring the level of rainwater in a tank that acts as an accumulator for an aqueduct. The embedded system relies on an Arduino MKR GSM 1400 board and an HC-SR04 ultrasonic sensor. For this application, memory has also been expanded thanks to an Arduino MKR SD Proto Shield, which allows storing data samples in case of connectivity issues. The basic operation principle of the device is shown in Figure 7.

Such as for the other embedded applications, there is a *setup* phase followed by a *loop* one. The only difference concerns the script associated with the resource in use. In this case, the application’s logic simply relies on a *filter* operation, based on which only samples whose values exceed one of two thresholds (above a maximum or below a minimum) are shipped to the cloud. As the tank is located in a wood area far from the landline, the system includes a GSM module installed in the board in order to send SMSs to notify the maintainer of the tank of the potential risk.

**Figure 8:**
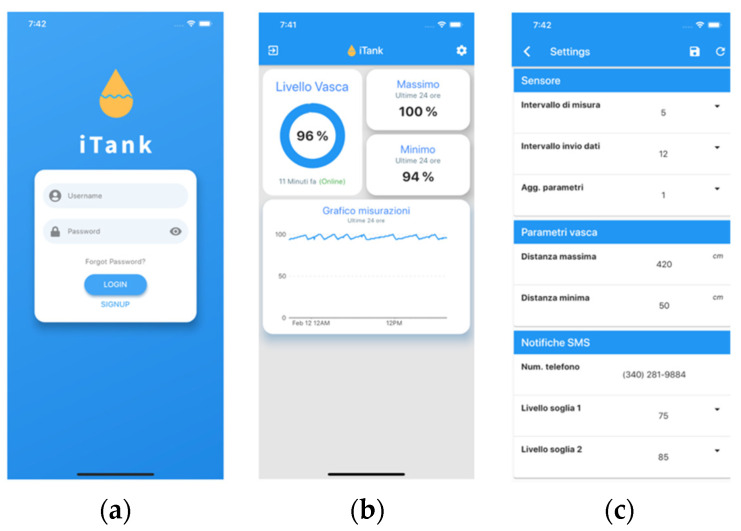
Snapshots from the tank level monitoring app: (**a**) login page; (**b**) tank monitoring info; (**c**) app settings.

In order to monitor and visualize the acquired data, a cross-platform mobile app (i.e., working on both iOS and Android devices) has been developed in Flutter, an open-source framework for building natively compiled, multi-platform applications from a single codebase [41]. The three main pages of the app are shown in Figure 8.

### 4.2. Environmental Use-Cases

A typical IoT application domain is given by environmental monitoring (e.g., [42,43,44,45]). This subsection presents two use-cases in this perspective. The first one monitors the air quality in a room, whereas the second allows checking the status of a plant and its surrounding environment.

#### 4.2.1. Air Quality Monitoring

This project concerns the realization of a system that monitors the concentration of harmful gases present in a closed environment, following an approach similar to the one proposed in [31], aiming at reducing the system complexity by taking into consideration only three toxic gases and monitoring them with a single sensor. The monitored gases are carbon monoxide (CO), nitrogen dioxide (NO_2_), and methane (CH_4_). The board used is an Arduino MKR WiFi 1010, whereas the gas sensor is a MiCS-6814, which communicates to the board through I2C. The application life-cycle is the same as in the previous cases. Data samples are periodically sent to the cloud (as specified in the script), together with the identifier of the measured type of gas.

**Figure 9:**
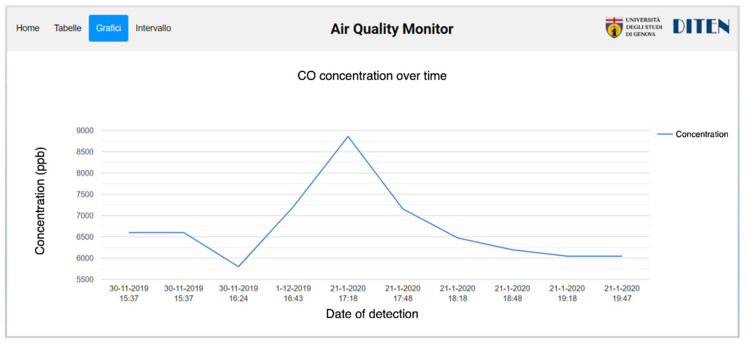
Measurements of the air quality system related to CO_2_ concentration in graphical format.

Similar to the other cases, a web page has been created to visualize data in graphical and tabular formats. Table 6 shows an example of carbon monoxide measurements, while Figure 9 displays a graphical view in which sample values are plotted over time.

#### 4.2.2. Plant Monitoring

**Figure 10:**
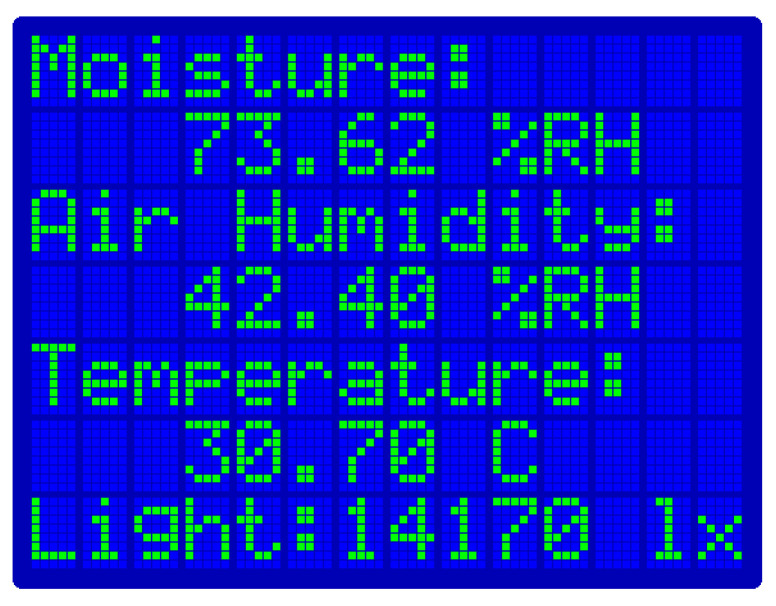
The plant monitoring system screen for local inspection.

This application aims at remotely monitoring plants and flowers. The chosen microcontroller is an Arduino Uno WiFi Rev 2, and three sensors are attached to it: a Sparkfun TSL2561 luminosity sensor, a DHT22 Pro v1.3 air-humidity and temperature sensor, and a DFRobot SEN0193 soil moisture capacitive sensor. The luminosity sensor provides data in Lux units and allows the user to know if the plant is receiving enough light, and the air-humidity and temperature sensor returns samples in °C and %RH, respectively, that give information regarding the environment where the plant is growing. Finally, the soil moisture sensor allows understanding whether the plant needs more water. In addition, a TFT screen has been installed on the Arduino device to visualize data directly from the source if necessary (Figure 10).

As the application involves heterogeneous measurements, Measurify records four features (one for each type of measurement), and the related scripts allow defining different sample times and processing steps for these different physical quantities.

**Figure 11:**
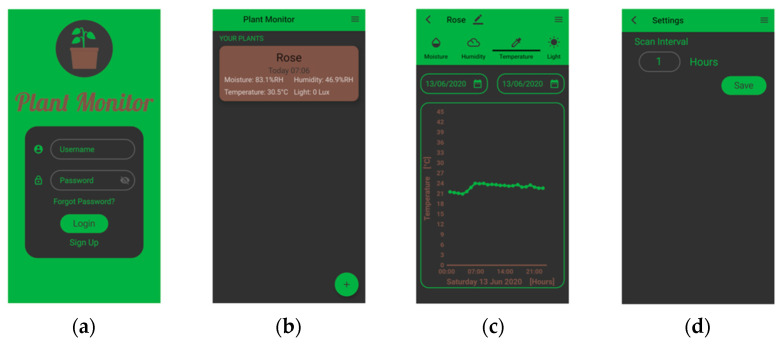
The plant monitoring app: (**a**) login page; (**b**) report on the monitored plants; (**c**) temperature graph over time; (**d**) scan interval settings.

As for other use-cases, in order to make data easier to interact with, a Flutter mobile app has been developed, namely Plant Monitor. The main pages of the app are shown in Figure 11. The app allows for an overview of the monitored plants but also graphs over time for each feature taken into consideration. Moreover, the scan interval can be set directly from the app. 

### 4.3. Sports Use-Cases

The level of miniaturization of edge devices has enabled applying digital technologies to sports activities by means of wearable devices (e.g., wristbands, cardio-bands, smartwatches, etc.), and devices that can be attached to sports tools (e.g., shoes, tennis rackets, motorbikes, cars, etc.) (e.g., [46,47,48,49,50]). This sub-section described two sports applications developed by applying the Edgine–Measurify platform.

#### 4.3.1. Smart Bike

This project aims at monitoring an enduro mountain bike (MTB) on a descent along a path in order to allow athletes to evaluate their performance. The following dimensions have been considered:

- Speed trend over time;
- Elevation profile of the route;
- Travel time;
- Maximum lean angle of the bike;
- Maximum slope of the route;
- Number of front fork compressions;
- Maximum speed reached.

**Figure 12:**
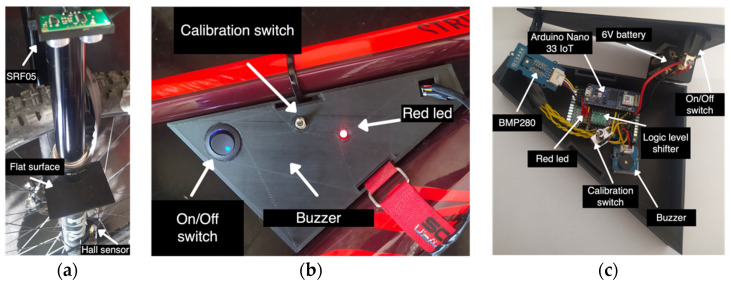
The smart bike main components: (**a**) the front fork with the ultrasonic sensor and the hall sensor; (**b**) the main core of the system, including a led and a buzzer to check if the system is running correctly; (**c**) the inside of the core, where the Arduino board is installed, and a battery powers the system.

In order to retrieve information about speed and time, a Grove–Hall sensor has been employed. This, attached to the front fork of the bike, allows counting the revolutions of the wheel so that speed, time, and space traveled can be deduced. For the number of front fork compressions, an ultrasonic sensor, the DFRobot SRF05, has been used. This, also installed on the fork, measures the distance from the front hub, which decreases when compressions occur. The elevation profile of the route has been measured through a Grove–Barometer BMP280, which detects the atmospheric pressure (in hPa). This measure is then converted to an altitude value as described in [51]. Lastly, to calculate the lean angle of the bike and the route slope, an accelerometer and a gyroscope were needed. Based on such requirements, it was decided to opt for an Arduino Nano 33 IoT board, which includes a built-in LMS6DS3 sensor, so that no other installation on the MTB was needed. Due to the presence of sensors operating at a voltage higher than 3.3 V (hall sensor and ultrasonic sensor work in the range 0–5 V) a logic level shifter, the Pololu 4-channel, was also employed to avoid system failures or damages. The final prototype, including the board and the sensors mounted on the MTB, is shown in Figure 12.

**Figure 13:**
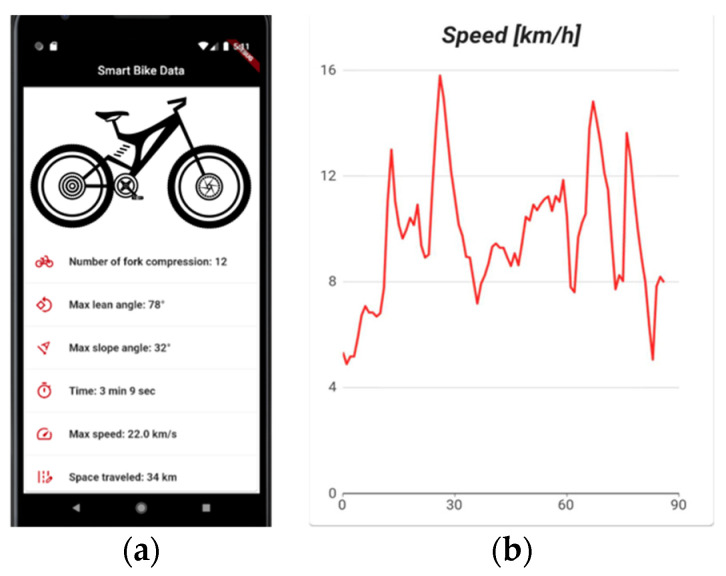
The smart bike app: (**a**) the bike data; (**b**) speed evolution during the tour.

In the execution loop, the edge system, after retrieving data samples from the sensors, connects to Measurify exploiting the Edgine library and, for each feature, sends the script-processed results to the cloud. By using the operations specified in Table 4, the scripts simply mandate the acquisition rate of the various signals and the expected computation (e.g., the maximum, within the specified sliding window). As in the previous use-cases, a Flutter mobile app has been developed. This allows the user to gather, in real-time, all the information about the smart bike and see informative graphs about the path traveled. Figure 13 shows the main page of the app along with a graph of the speed evolution during a tour.

#### 4.3.2. Smart Racket

The last application example in the sports area concerns the realization of a tennis racket support to collect data about an athlete’s strokes. The rationale of this project is bound to the need for supporting both professional, amateur, and novice tennis players through collection of raw data and elaboration of statistics during workouts and matches. 

The work, which is in progress, involves the implementation of a system able to measure speed, rotation, and point and angle of impact in order to allow distinguishing the various types of strokes. Six different types of strokes have been considered:

- Serve;
- Forehand groundstroke;
- Backhand groundstroke;
- Overhead smash;
- Forehand volley;
- Backhand volley.

Accelerometer and gyroscope sensors are needed for this purpose. Since the Arduino Nano 33 IoT board has a built-in LMS6DS3 sensor, we chose it as the development board. The sensor raw data themselves are not sufficient to distinguish among the six types of strokes. Therefore, the first goal of the application is to support the creation of a dataset for each class. Then, a neural model will be trained with this dataset to recognize the strokes following an edge learning approach [52]. 

The proposed method aims at improving the work done in [53], where a motion sensor is attached to the racket to classify the stroke type between three classes: serve, groundstroke, and volley. Additionally, for the serve stroke, a regression model is proposed to estimate the ball speed, whereas two other models are proposed for groundstroke and volley: a regression and a physical one. The physical model is best suited for skilled players who have constant stroke gestures, while the regression model is more appropriate for beginners, who have varying stroke gestures. 

**Figure 14:**
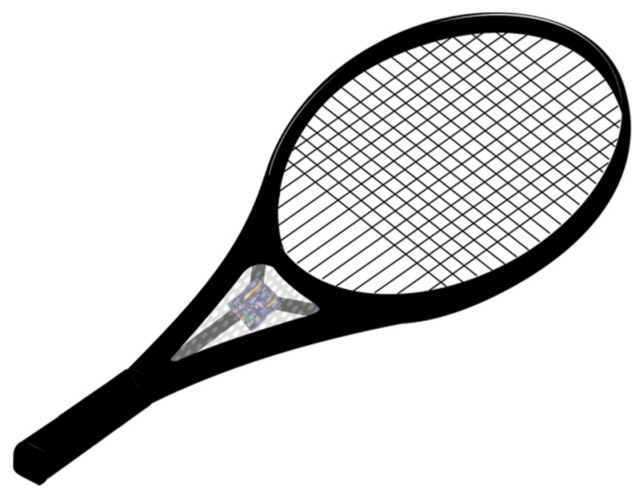
Outlook of a smart racket.

The Edgine system is expected to process data locally and send to the Measurify information needing minimal post-processing. A mobile app will be developed to show, in real-time, aggregate data of a tennis match obtained through scripts ad hoc created for the resource (e.g., number of forehand groundstrokes, maximum serve speed, percentage of forehand groundstrokes compared to backhand groundstrokes, etc.). This will require interfacing Edgine with the outcomes of an embedded neural network classifying the raw sensors’ time-series into the six mentioned classes. The final prototype is expected to have a structure similar to that shown in Figure 14. Due to the reduced dimension of the racket handle, the board will be inserted in the throat of the racket, protected by a pitted case. 

## 5. Discussion

Table 7 provides a quantitative outlook of the six use-cases presented in the previous section. It appears that a whole end-to-end IoT application, featuring an edge device, a cloud installation, and a client (web/mobile app), is regularly built in less than one man month, by a third year Electronic Engineering BSc student. The time includes familiarization with the framework. This has allowed the students (i.e., the application designers) to focus on the application requirements, which is of key importance for a proper application design. There is a variance due to the application complexity, which is typically reflected by the number of sensors to use and features to measure. However, the value of the variance is limited, and the development time is quite predictable. This is important for an Engineering BSc thesis project, but also in the business field. While the sample of students is limited, we argue that they are representative of a normal knowledge level at the end of an Information Technology Engineering BSc course. 

According to the students directly involved in the thesis projects, the benefits of using the Measurify framework for developing an end-to-end IoT application were manifold. First, the ease of use and installation of the system allowed the developer to focus on dataflow design rather than on the aspects of cloud interfacing, thus considerably reducing the development time. Furthermore, the possibility of preparing scripts chaining a limited set of instructions made it possible to achieve the projects’ objectives in a logical and precise way.

The main issues encountered concerned the need for the developer to hardwire the SSID credentials in the code and a certain difficulty in understanding the interaction of the edge system with the cloud. In particular, since the HTTPS requests made by Edgine are hidden within the library, there is no immediate feedback for the user regarding the successful storage of data in the cloud server. This stage of understanding the framework’s functioning and preparing it for the intended purpose has significantly lengthened development times, but the elementary professional level of the subjects must also be taken into consideration.

Comparing the achieved results with the state-of-art case studies reported in the Related work section, we can sum up strengths and weaknesses of the presented system. The Edgine–Measurify framework is characterized by a clear versatility, which is due to its powerful design abstractions, and it allows the developers to manage heterogeneous use-cases while maintaining the same system architecture. This has clear implications on application robustness, as the applications exploit a widely used and tested (in various contexts) generic system. 

In the workflow supported by the system, for creating a new applications, it suffices to configure a Measurify installation (device and feature resources, plus relevant tags, if needed), along with the script(s) needed in the edge environment. The exploitation of non-cloud-vendor-locked software makes the system portable across cloud systems. Edgine is also platform-independent, which makes the system work on a large variety of development boards and OSs, and it can also be used in a simulated environment [35]. On the other hand, the main limitations of the current release concern the restricted operations pool (along with the lack of support for on-the-edge ML inference) and the inability of the *send* operation to manage multidimensional data, which restricts the operational field of the system.

## 6. Conclusions

As IoT technologies are increasing the capability of collecting huge quantities of data from the field, the availability of tools for creating new, data-rich applications is becoming ever more relevant. In our view, such tools should be characterized by wide usability, in terms of (i) independence of the specific edge/cloud platform, (ii) open-source availability, and (iii) seamless and effective applicability in a variety of IoT domains.

In this manuscript, we assessed the versatility of an end-to-end system featuring a measurement-oriented framework for managing smart things (namely, Measurify, which is available open-source at: measurify.org, accessed on 27 December 2021). The framework, accessible through RESTful APIs, exposes to developers a set of resources that can be used in very different contexts. 

Our experience with BSc thesis projects in the last academic year has shown the potential of such a system—both from a didactic and a professional point of view—and helped us to identify strengths and weaknesses.

The design abstractions in Measurify and Edgine (generic sensor configuration, simple language with chainable operations for processing time-series on the edge, seamless WiFi/GSM communication) allowed developers to be productive and focus on the actual application requirements and the high-level design choices to define the edge system (microcontroller and its sensors), skipping the huge set-up times needed to start a solution from scratch. Moreover, robustness of the resulting application strongly benefits from the exploitation of an extensively tested framework. The experience also highlighted some usability issues, particularly concerning the network connection specification and the lack of user feedback about the data upload to the server.

New releases of the framework will include a credential retrieving function from an encrypted file (at the moment, WiFi credentials are put in plain text in the main function). A significant extension will concern the support of the execution on the edge of ML models, that will be dynamically configurable from the cloud, according to the Measurify paradigm. These models will be handled with new specific operations of the script language. In addition, real-time estimations of system memory usage and power consumption will be provided through the interface of the Edgine script language, so to allow monitoring the functional behavior of the system. Finally, support for the delivery of more complex data types is foreseen. At present, the *send* operation only supports 1D float samples, while multi-dimensionality would allow more efficient data preparation and transfer and bulk sending of more complex data types (e.g., images) as well.

Concerning user assessment, further qualitative and quantitative experiments should be performed in two main directions. One involves professional users, thus considering more complex projects, also possibly implying an extension of the operation set, as anticipated above. A further direction involves tests with engineering students in order to better understand the didactical value and implications of the tool.

## Figures and Tables

**Table 1:** Outlook of edge-computing case-studies.

Ref.	Application Domain(s)	Edge Device	Sensors	Cloud Services	Publicly Available	Edge ML Support
[27]	Climatic enclosures	Jetson Nano	Temperature, humidity, light, soil moisture	-	✕	✓
[29]	Firefighting	Intel Fog Reference Design	Wearables, infrared camera, toxic gas, camera	Amazon EC2	✕	✓
[30]	Video surveillance	-	Camera	Microsoft Cognitive Services	✕	✕
[31]	Air pollution monitoring	Arduino	Gas, temperature, humidity	IBM Cloud	✕	✕
[33]	Manufacturing active maintenance	Raspberry Pi	-	Private cloud	✕	✓
[34]	Multiple: health, buildings, self-driving cars	Simulated generic IoT device	Multiple virtual sensors	IoTSim-Edge	✓	✕

**Table 2:** Outlook of the main Measurify resources.

Element	Description
Measurement	Value of a feature measured by a device for a specific thing.
Thing	A generic object target of a measurement (i.e., within which a measurement is performed).
Device	A sensor that provides measurements of a thing.
Feature	A physical dimension measured by a device.
Script	A JSON string that contains information on how to manipulate, store, and transmit streams of measurements coming from devices. This is the program to be executed by a field device.
Tag	Labels attachable to resources, to better specify them.
Users	Users (with roles and rights) that have access to the resources of the current application.

**Table 3:** HTTP requests performed by Edgine.

Request Type	Object	Description
POST	Login credentials	Login into the cloud, JWT is received as a response.
GET	Resource description and executable script(s)	Description of the in-use resource and executable script(s) are retrieved from the cloud.
POST	Measurements	Edge-processed data are shipped to the cloud.

**Table 4:** Operations currently available in the Edgine script language.

Operation	Description
Send	Sends to the API all the elements of the data stream.
Map	A new data stream is created by performing a simple arithmetic operation between two operands.
Max/min	A new data stream is created containing only the min/max value among the values in the input stream.
Window/sliding window	A new data stream is created by applying a two-operand function on an accumulator, initialized to the value of the second argument, and on each input element, for a number of values indicated by the size of the window/sliding window.
Filter	A new data stream is created using only the elements of its input stream that have a value within a specified range.
Average/median/standard deviation	A new data stream is created by taking the average/median/standard deviation of a specified number of samples in its input stream.

**Table 5:** Report of the five most recent shocks occurred.

Time	Magnitude	Angle XY	Angle YZ	Angle XZ
12 September 2021, 16:45:50	3	251	230	254
12 September 2021, 16:45:50	6	270	185	268
12 September 2021, 16:45:50	3	92	353	252
12 September 2021, 16:45:50	3	93	341	260
12 September 2021, 16:45:50	4	67	287	277

**Table 6:** Measurements of the air quality system related to CO concentration in tabular format.

Date	Time	Concentration (ppb)
21 January 2020	19:47	6044.16
21 January 2020	19:18	6044.16
21 January 2020	18:48	6193.68
21 January 2020	18:18	6472.43
21 January 2020	17:48	7155.01
21 January 2020	17:18	8858.53

**Table 7:** Quantitative observation of the presented use-cases.

Use-Case	Edge Device	No. Sensors	No. Features	Measurify Configuration (h)	Edgine (h)	Client Development (h)
Shock monitoring	Arduino MKR WiFi 1010	1	4	35	50	30
Tank level monitoring	Arduino MKR GSM 1400	1	1	30	40	40
Air quality monitoring	Arduino MKR WiFi 1010	1	3	35	50	25
Plant monitoring	Arduino Uno WiFi Rev 2	3	4	40	50	40
Smart bike	Arduino Nano 33 IoT	4	6	45	60	35
Smart racket	Arduino Nano 33 IoT	1	6	in progress	in progress	in progress

## Data Availability

The Measurify framework, including the Edgine module, is available at https://github.com/measurify, accessed on 27 December 2021.
